# Association of Retinal Nerve Fiber Layer Thickness With Brain Alterations in the Visual and Limbic Networks in Elderly Adults Without Dementia

**DOI:** 10.1001/jamanetworkopen.2018.4406

**Published:** 2018-11-09

**Authors:** Juan Luis Méndez-Gómez, Amandine Pelletier, Marie-Bénédicte Rougier, Jean-François Korobelnik, Cédric Schweitzer, Marie-Noëlle Delyfer, Gwenaëlle Catheline, Solène Monfermé, Jean-François Dartigues, Cécile Delcourt, Catherine Helmer

**Affiliations:** 1 University Bordeaux, Institut National de la Santé et de la Recherche Médicale (INSERM), Bordeaux Population Health Research Center, Unité Mixte de Recherche (UMR) 1219, Bordeaux, France; 2 Centre National de la Recherche Scientifique (CNRS), Institut de Neurosciences Cognitives et Intégratives d’Aquitaine (INCIA), UMR 5287, Bordeaux, France; 3 Ophthalmology, University Hospital, Bordeaux, France; 4 École Pratique des Hautes Études (EPHE), Paris Sciences et Lettres (PSL) Research University, Bordeaux, France; 5 Memory Consultation, Centre Mémoire de Ressource et de Recherche (CMRR), University Hospital, Bordeaux, France; 6 Clinical Epidemiology Unit, INSERM, Centre d’Investigation Clinique (CIC) 1401, Bordeaux, France

## Abstract

**Importance:**

The eye is a sensory organ that is easily accessible for imaging techniques, allowing the measurement of the retinal nerve fiber layer (RNFL) thickness. The eye is part of the central nervous system, and its neurons may be susceptible to degeneration; therefore, changes in the RNFL thickness may reflect microstructural and volume alterations in the brain.

**Objective:**

To explore the association between the peripapillary RNFL thickness and brain alterations in the visual and limbic networks in elderly people without dementia.

**Design, Setting, and Participants:**

Cross-sectional analysis of the Three-City/Antioxydants, Lipides Essentiels, Nutrition et Maladies Oculaires (Alienor) Study cohort (April 2009 to December 2010). The dates of analysis were July 2017 to August 2018. The setting was a population-based study in France. The brain volume analysis included 104 participants, and the diffusion tensor imaging analysis included 79 participants.

**Main Outcomes and Measures:**

Global RNFL was assessed by spectral-domain optical coherence tomography. Brain volumes were assessed via T1-weighted magnetic resonance imaging by measurement of the global white and gray matter fractions and the hippocampal fraction. Brain microstructural alterations were assessed with diffusion tensor imaging at the level of the posterior thalamic radiations, the limbic system tracts (the fornix and cingulum bundles), and the posterior limb of the internal capsule (control region). Linear regression models adjusted for several confounders were performed.

**Results:**

Among a total of 104 participants, the mean (SD) age was 80.8 (3.9) years, and the cohort was 56.7% women (n = 59). The mean (SD) global RNFL thickness was 89.3 (12.9) µm. A thicker RNFL was associated with a greater hippocampal fraction (quantity of increase β = 0.013; 95% CI, 0.001-0.025 per 10-μm increase in the RNFL thickness) and better diffusion tensor imaging variables in the global cingulum (mean diffusivity β = −0.007; 95% CI, −0.015 to −0.000) and the hippocampal part of the cingulum (mean diffusivity β = −0.009; 95% CI, −0.016 to −0.002 and radial diffusivity β = −0.010; 95% CI, −0.018 to −0.002) and the posterior thalamic radiations (fractional anisotropy β = 0.008; 95% CI, 0.000-0.017). No significant associations were found with other magnetic resonance imaging volumes or with other diffusion tensor imaging variables. In particular, there was no significant association with the control region of interest.

**Conclusions and Relevance:**

Results of this study suggest that in elderly individuals without dementia, a thicker RNFL was associated with better magnetic resonance imaging variables both in a region that included the visual pathways and in regions particularly involved in the neurodegenerative processes of Alzheimer disease.

## Key Points

**Question:**

Is the peripapillary retinal nerve fiber layer (RNFL) thickness correlated with brain alterations in regions that are vulnerable to neurodegenerative processes?

**Findings:**

Among 104 elderly people with dementia in this population-based study, a thicker RNFL was associated with a higher hippocampal fraction and better diffusion tensor imaging variables in the global and hippocampal part of the cingulum, 2 regions closely associated with the neurodegenerative processes of Alzheimer disease.

**Meaning:**

The assessment of axonal thickness in the retina, which is a quick measurement to perform, may provide some elements of brain magnetic resonance imaging abnormalities at an early stage of neurodegeneration.

## Teaser

This population-based, cross-sectional study explores the association between the peripapillary retinal nerve fiber layer thickness and brain alterations in the visual and limbic networks in elderly people without dementia.

## Introduction

Because of their common embryologic origin, the retinal nerve fiber layer (RNFL) is anatomically connected with the central nervous system; therefore, the eye is a sensory organ that is part of the central nervous system, with neurons that may be susceptible to direct or indirect degeneration.^1^ Using advanced imaging techniques, such as spectral-domain optical coherence tomography (SD-OCT), provides easy and quick access to accurately evaluate the RNFL, which may reflect neurodegenerative changes in the brain.^2^

Previous studies have reported RNFL thinning in patients with Alzheimer disease (AD) and mild cognitive impairment (MCI) vs healthy controls; these results were confirmed in 2 recent meta-analyses,^3,4^ revealing significantly reduced mean RNFL thickness in AD and MCI. Moreover, within a large population, the Rotterdam Study^5^ recently found that a thinner RNFL was associated with a higher risk of developing dementia and AD in subsequent years, suggesting that a thinner RNFL may be a biomarker for dementia and AD. Nevertheless, beyond the clinical diagnosis of AD and MCI, the relationship between the RNFL and brain structures remains underexplored in the elderly population. Indeed, previous imaging studies have explored the connections between the RNFL and brain alterations observed in specific pathologies, such as multiple sclerosis^6,7,8,9,10^ and glaucoma.^11^ However, to our knowledge, only 2 previous studies have explored this association beyond these specific pathologies. The first, a population-based study^12^ that included 164 elderly people who were mainly cognitively impaired without dementia showed that a reduced RNFL thickness was associated with a reduced gray matter (GM) volume in the temporal lobe. The second study^13^ included 79 neurologically healthy older adults and found an association between RNFL thinning and smaller mediotemporal lobe volumes. However, these 2 studies analyzed only the brain volume. Yet, other brain imaging techniques, such as diffusion tensor imaging (DTI), can be used to assess the white matter (WM) microstructural integrity, revealing another aspect of the neurodegenerative process. Moreover, several studies since 2006 indicate that DTI variable measurement is a sensitive and powerful method, showing global age-related modifications^14^ and fine modifications of specific networks, such as the limbic system.^15,16^

We hypothesized that changes in the RNFL thickness may reflect microstructural and volume alterations in the brain, not only in the visual pathways but also in regions that are particularly vulnerable to the neurodegenerative processes occurring in AD and/or MCI. Therefore, within a population-based study of elderly people in France, we aimed to explore the associations between the peripapillary RNFL thickness and brain alteration measures using anatomical images to assess the global and hippocampal volumes and using diffusion-weighted images to extract variables in specific WM bundles in the posterior thalamic radiations (including the optic radiations), the WM connecting tracts of the limbic system (ie, the fornix and cingulum bundles), and the posterior limb of the internal capsule as a control region.

## Methods

### Study Population

Our study population is from the Three-City Study,^17^ a prospective population-based cohort of French adults 65 years or older that aims to estimate the risk of dementia and cognitive impairment attributable to vascular factors in 3 cities in France (Bordeaux, Dijon, and Montpellier). Among the initial 9294 participants, our study focused on those from the Bordeaux site; 2104 participants were included at baseline (1999-2001) and subsequently reexamined 2, 4, 7, 10, and 12 years later. Data regarding their sociodemographic characteristics, lifestyle, physical and mental health, medications, disabilities, and cognitive function were assessed at baseline and at each follow-up. At the 10-year follow-up, a brain imaging examination was proposed; 239 participants agreed to undergo this examination.

At the 7-year follow-up (in 2006-2007), 963 participants agreed to participate in the Antioxydants, Lipides Essentiels, Nutrition et Maladies Oculaires (Alienor) Study,^18^ consisting of an ophthalmological examination. These participants were then followed up every 2 years with eye examinations that were concurrent with the Three-City Study follow-ups. In 2009-2010, SD-OCT examination of the retina and optic nerve was included in the eye examination.^19^ Our study sample consisted of people with valid peripapillary RNFL measurements obtained in April 2009 to December 2010, and valid brain magnetic resonance imaging (MRI) (without any major cerebral pathologies) obtained at the same time (Figure). The dates of analysis were July 2017 to August 2018. People with prevalent dementia, which was systematically screened for and diagnosed on the *Diagnostic and Statistical Manual of Mental Disorders* (Fourth Edition) criteria^20^ using a 3-step standardized procedure previously described,^17^ were excluded. We also excluded people with a medical history of stroke, as well as those with eye diseases affecting the RNFL measurement (glaucoma, vitreomacular traction, myopic chorioretinopathy, peripapillary choroidal neovascularization, or myelinated retinal nerve fibers). In addition, because the ocular axial length (AL) is associated with the RNFL thickness,^21^ we also excluded people with missing values for AL. Finally, our study sample included 104 participants for the brain volume analysis and 79 participants for the DTI analysis. Compared with the total Three-City Study population, our study sample was older, included more women, had a lower educational level, had more hypertension, and had a lower Mini-Mental State Examination score (eTable in the Supplement).

**Figure.**
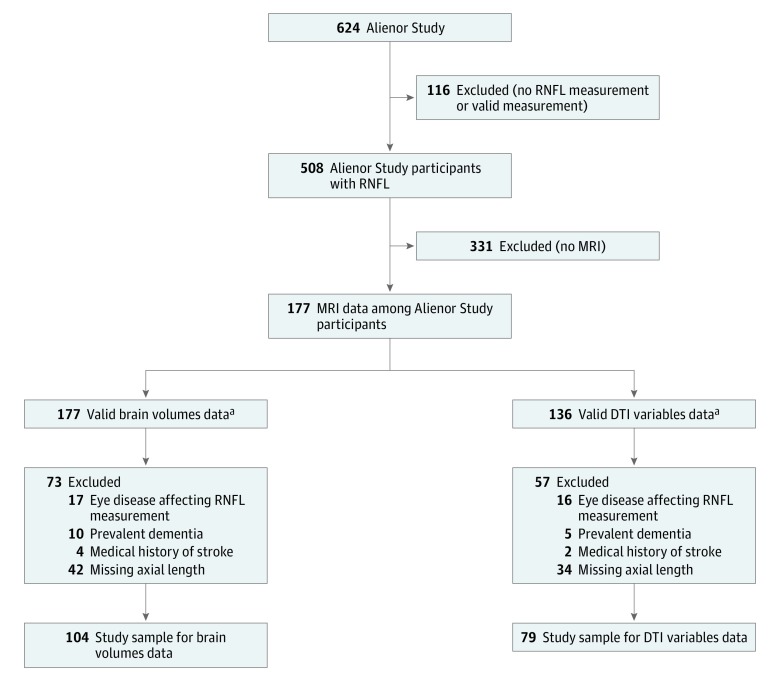
Flowchart of the Selected Participants Alienor indicates Antioxydants, Lipides Essentiels, Nutrition et Maladies Oculaires; DTI, diffusion tensor imaging; MRI, magnetic resonance imaging; and RNFL, retinal nerve fiber layer. ^a^Valid MRI images without the presence of tumors or major cerebrovascular pathologies and showing good-quality processes.

Ethics committee approvals were obtained from the ethical committee of Bordeaux for the Alienor Study and from the ethical committees of the university hospitals of Le Kremlin-Bicêtre and Sud-Méditerranée III for the Three-City Study. Each participant signed an informed consent form. We followed the Strengthening the Reporting of Observational Studies in Epidemiology (STROBE) reporting guideline for observational studies to guide the reporting of this study.

### MRI Assessment of Brain Structure

#### MRI Acquisition

The MRI examinations were performed using a 3-T imaging system (Achieva; Philips Medical Systems) equipped with an 8-channel head coil (SENSE; Philips Medical Systems). For each person, anatomical high-resolution MRI volumes were acquired in a transverse plane using a 3-dimensional (3-D) magnetization-prepared rapid gradient echo (MPRAGE) T1-weighted sequence, and diffusion-weighted images were acquired using a single-shot echo-planar sequence (21 noncollinear directions and b value of 1000 s/mm^2^).

#### MRI Processing

##### Brain Volumes

Global brain volumes were assessed using voxel-based morphometry^22,23^ implemented in the VBM8 toolbox (http://dbm.neuro.uni-jena.de/vbm/) within SPM8 software (https://www.fil.ion.ucl.ac.uk/spm/software/spm8/). Regarding the hippocampal volume, segmentations and volume estimations were performed using FIRST,^23^ a component of Functional Magnetic Resonance Imaging of the Brain (FMRIB) Software Library. More details have been reported previously.^24^ The right and left hippocampal volumes were averaged for the statistical analyses. The hippocampal fraction (HF) (the hippocampal volume divided by the total intracranial volume [TIV]) was used as an index of hippocampal atrophy. Similarly, our analyses included the GM fraction (the GM volume divided by the TIV), the WM fraction (the WM volume divided by the TIV), and the brain parenchymal fraction (BPF) (GM plus WM volumes divided by the TIV).

##### DTI Variables

The integrity of WM microstructure was examined using DTI. The DTI variables evaluate both the directionality and the magnitude of water diffusion in brain tissue. Within WM, the diffusion of water is restricted along bundles.^25^ Fractional anisotropy (FA) represents the degree of the directionality of water diffusivity along the fibers; axial diffusivity (AxD) and radial diffusivity (RD) quantify the magnitude of diffusion along the principal and perpendicular directions of the fibers, respectively; and mean diffusivity (MD) represents a global measure of diffusion. Therefore, higher directionality, as indicated by FA, and lower magnitude of diffusion, as indicated by diffusivity variables (ie, AxD, RD, and MD), generally indicate preserved microstructure. We evaluated the DTI variables in the following areas: (1) the posterior thalamic radiations, including the optic tracts, and (2) the bundles of the limbic system, which are thought to be altered prematurely in AD processes (ie, the fornix and cingulum bundles). The latter 2 bundles were analyzed both globally and specifically in their part running along the hippocampus (as defined in the Johns Hopkins University atlas,^26^ the stria terminalis of the fornix and the hippocampal cingulum). We also analyzed a control region of interest, the posterior limb of the internal capsule (including the motor bundles), which was not specifically involved in either the visual pathway or AD.

### SD-OCT Measures

Participants underwent a retinal SD-OCT examination without pupil dilation using a software program (SPECTRALIS, version 5.4.7.0; Heidelberg Engineering) and performed by the same experienced technician. The SD-OCT offers a detailed cross-sectional image of the retina.^27^ The RNFL thickness was acquired using the following conditions^19^: high-speed resolution mode, circle diameter of 3.5 mm, size *x* of 768 pixels (10.9 mm), size *z* of 496 pixels (1.9 mm), scaling *x* of 14.14 lumens per pixel, scaling *z* of 3.87 lumens per pixel, and automatic real-time (ART) mode of 16 images. The minimum reliable value of the global thickness was retained when a reliable measurement was obtained in both eyes; otherwise, only the measurement from the eye with a reliable value was retained.

### Other Variables

The covariates considered were age, sex, educational level, *APOE4* genotype, diabetes (antidiabetic treatment and/or a fasting glucose level ≥126 mg/dL [to convert to millimoles per liter, multiply by 0.555] or a nonfasting glucose level ≥198 mg/dL), hypertension (blood pressure >140/90 mm Hg or antihypertensive medication), tobacco consumption, cataract surgery, vertical diameter of the optic nerve disc (the mean of 2 measurements), and AL. Axial length was assessed for all participants via anatomical high-resolution MRI images in a transverse plane based on a 3-D MPRAGE T1-weighted sequence.^28^ Two operators (J.L.M.-G. and S.M.) separately measured the AL, and the mean of 2 measurements was retained. In case of a difference exceeding 2 mm between the 2 values, a third measurement was made, and the 2 closer measurements were retained for the mean. This procedure was validated in a subsample of 79 participants with available AL measurements obtained with noncontact partial coherence laser interferometry (IOL Master; Carl Zeiss Meditec AG); these latter correlated strongly with the AL measurements from the MRI images (Pearson product moment correlation *r* = 0.89, *P* < .001).

### Statistical Analysis

Descriptive statistics were calculated for each MRI subsample (brain volume analysis and DTI analysis). Multiple linear regression models were performed to analyze the association between the RNFL and MRI variables. The β coefficients, which correspond to the quantity of increase in brain variables (either brain volumes or DTI variables) per 10-μm increase in the RNFL thickness, and their 95% CIs are provided. The models were adjusted for ocular variables, age, sex, educational level, *APOE4* genotype, diabetes, hypertension, and AL. The distribution of the residuals in the models was examined graphically to test the assumption of normal distribution. All statistical analyses were performed with a software program (SAS, version 9.3; SAS Institute Inc), and *P* < .05 (2-sided) was considered significant.

## Results

The population characteristics are listed in Table 1. Among a total of 104 participants, the mean (SD) age was 80.8 (3.9) years, and the cohort was 56.7% women (n = 59). Among the participants, 16.0% (16 of 100) were *APOE4* carriers, 8.7% (9 of 104) had diabetes, 73.1% (76 of 104) had hypertension, 34.6% (36 of 104) were smokers, and 40.4% (42 of 104) had undergone cataract surgery. The mean (SD) global RNFL thickness was 89.3 (12.9) µm.

**Table 1.** Characteristics of the Study Participants

Variable	Brain Volumes (n = 104)^a^	DTI Variables (n = 79)^b^
Age, mean (SD), y	80.8 (3.9)	80.4 (3.8)
Female, No. (%)	59 (56.7)	45 (57.0)
Educational level, No. (%)		
Elementary school	9 (8.7)	6 (7.6)
Short secondary school	40 (38.5)	34 (43.0)
Higher level	55 (52.9)	39 (49.4)
*APOE4* genotype, No./total No. (%)	16/100 (16.0)	12/76 (15.8)
Diabetes, No. (%)	9 (8.7)	7 (8.9)
Hypertension, No. (%)	76 (73.1)	57 (72.2)
Smokers, No. (%)	36 (34.6)	25 (31.6)
Cataract surgery, No. (%)	42 (40.4)	32 (40.5)
Vertical diameter of the optic nerve disc, mean (SD), mm	2.0 (0.2)	2.1 (0.2)
Axial length, mean (SD), mm	23.7 (1.1)	23.7 (1.2)
Global RNFL thickness, mean (SD), μm	89.3 (12.9)	89.2 (13.2)
MMSE score, mean (SD)	27.7 (2.2)	27.8 (2.1)
Brain volumes, mean (SD), % of TIV		
BPF	76.1 (2.4)	NA
HF	0.49 (0.07)	NA
WM fraction	35.7 (2.2)	NA
GM fraction	40.3 (2.5)	NA
DTI variables, mean (SD)		
Global FA	NA	0.46 (0.02)
Global AxD, 1000 s/mm^2^	NA	1.21 (0.03)
Global RD, 1000 s/mm^2^	NA	0.56 (0.03)
Global MD, 1000 s/mm^2^	NA	0.78 (0.03)

Abbreviations: AxD, axial diffusivity; BPF, brain parenchymal fraction; DTI, diffusion tensor imaging; FA, fractional anisotropy; GM, gray matter; HF, hippocampal fraction; MD, mean diffusivity; MMSE, Mini-Mental State Examination (score range, 0-30; higher scores indicate better cognition); NA, not applicable; RD, radial diffusivity; RNFL, retinal nerve fiber layer; TIV, total intracranial volume; WM, white matter.

^a^ Missing data for brain volumes: 4 for *APOE* genotype, 3 for vertical diameter of the optic nerve disc, 1 for MMSE score, and 7 for HF.

^b^ Missing data for DTI variables: 3 for *APOE* genotype, 3 for vertical diameter of the optic nerve disc, and 1 for MMSE score.

Regarding the brain volumes, the mean (SD) BPF was 76.1% (2.4%), and the mean (SD) HF was 0.49% (0.07%). Regarding the DTI variables, the mean (SD) global FA was 0.46 (0.02), and the mean (SD) global MD was 0.78 (0.03).

### RNFL Thickness and Brain Volumes

The multivariable analysis identified a significant association between the RNFL thickness and the hippocampus: a thicker RNFL was associated with a greater HF (quantity of increase β = 0.013; 95% CI, 0.001-0.025 per 10-μm increase in the RNFL thickness; *P* = .03) (Table 2). No significant associations were found between the RNFL and other MRI volume measures (ie, BPF and global GM and WM fractions).

**Table 2.** Associations Between the Global RNFL Thickness and Brain Volumes Among 97 Participants^a^

Variable, % of TIV	β (95% CI)	*P* Value
BPF	0.174 (−0.202 to 0.551)	.36
HF^b^	0.013 (0.001 to 0.025)	.03
WM fraction	0.051 (−0.324 to 0.425)	.79
GM fraction	0.123 (−0.245 to 0.492)	.51

Abbreviations: BPF, brain parenchymal fraction; GM, gray matter; HF, hippocampal fraction; RNFL, retinal nerve fiber layer; TIV, total intracranial volume; WM, white matter.

^a^ Seven brain volumes participants who had missing values for at least 1 covariate were excluded from the multivariable analysis. The multiple linear regression models were adjusted for age, sex, educational level, *APOE* genotype, diabetes, hypertension, tobacco consumption, cataract surgery, vertical diameter of the optic nerve disc, and axial length. The β coefficients are presented for a 10-μm increase in the RNFL thickness.

^b^ Among 90 participants.

### RNFL Thickness and DTI Variables

The multivariable analysis showed significant associations between the RNFL thickness and regions that include the visual pathway and regions implicated in AD-associated neurodegenerative processes (Table 3). A thicker RNFL was associated with a higher FA (reflecting better integrity of WM bundles) (β = 0.008; 95% CI, 0.000-0.017 per 10-μm increase in the RNFL thickness; *P* = .045) in the posterior thalamic radiations. A thicker RNFL was also associated with a lower MD in the global cingulum (β = −0.007; 95% CI, −0.015 to −0.000; *P* = .04), as well as a lower MD (β = −0.009; 95% CI, −0.016 to −0.002; *P* = .02) and a lower RD (β = −0.010; 95% CI, −0.018 to −0.002; *P* = .02) in the hippocampal part of the cingulum. There were no significant associations with other DTI variables in the regions explored. In particular, there was no significant association with the control region of interest (the posterior limb of the internal capsule).

**Table 3.** Associations Between the Global RNFL Thickness and DTI Variables Among 73 Participants^a^

Variable	β (95% CI)	*P* Value
**FA**		
Global WM	0.002 (−0.002 to 0.005)	.38
Entire fornix	−0.003 (−0.014 to 0.008)	.57
Entire cingulum	0.005 (−0.001 to 0.012)	.12
Hippocampal part of the fornix	0.002 (−0.004 to 0.008)	.57
Hippocampal part of the cingulum	0.007 (−0.001 to 0.001)	.07
Posterior thalamic radiations	0.008 (0.000 to 0.017)	.045
Posterior limb of the internal capsule	0.001 (−0.004 to 0.005)	.75
**AxD**		
Global WM	−0.002 (−0.007 to 0.003)	.44
Entire fornix	0.006 (−0.018 to 0.031)	.60
Entire cingulum	−0.007 (−0.017 to 0.003)	.17
Hippocampal part of the fornix	−0.008 (−0.020 to 0.005)	.23
Hippocampal part of the cingulum	−0.006 (−0.019 to 0.007)	.34
Posterior thalamic radiations	−0.001 (−0.015 to 0.012)	.85
Posterior limb of the internal capsule	0.001 (−0.009 to 0.011)	.83
**MD**		
Global WM	−0.003 (−0.008 to 0.003)	.32
Entire fornix	0.011 (−0.024 to 0.046)	.53
Entire cingulum	−0.007 (−0.015 to −0.000)	.04
Hippocampal part of the fornix	−0.005 (−0.015 to 0.005)	.33
Hippocampal part of the cingulum	−0.009 (−0.016 to −0.002)	.02
Posterior thalamic radiations	−0.008 (−0.019 to 0.003	.18
Posterior limb of the internal capsule	0.000 (−0.005 to 0.005)	.98
**RD**		
Global WM	−0.003 (−0.009 to 0.003)	.32
Entire fornix	0.013 (−0.028 to 0.055)	.52
Entire cingulum	−0.008 (−0.016 to 0.000)	.06
Hippocampal part of the fornix	−0.004 (−0.014 to 0.007)	.51
Hippocampal part of the cingulum	−0.010 (−0.018 to −0.002)	.02
Posterior thalamic radiations	−0.011 (−0.023 to 0.001)	.08
Posterior limb of the internal capsule	−0.001 (−0.005 to 0.004)	.85

Abbreviations: AxD, axial diffusivity; DTI, diffusion tensor imaging; FA, fractional anisotropy; MD, mean diffusivity; RD, radial diffusivity; RNFL, retinal nerve fiber layer; WM, white matter.

^a^ Six DTI variables participants who had missing values for at least 1 covariate were excluded from the multivariable analysis. Multiple linear regression models were adjusted for age, sex, educational level, *APOE* genotype, diabetes, hypertension, tobacco consumption, cataract surgery, vertical diameter of the optic nerve disc, and axial length. The β coefficients are presented for a 10-μm increase in the RNFL thickness. The DTI variables are expressed in 1000 s/mm^2^.

## Discussion

In an elderly population without dementia, we found that a thicker global peripapillary RNFL was associated with better DTI variables in the posterior thalamic radiations, which include the visual pathway. In addition, a thicker RNFL was also associated with a higher HF and better DTI variables in the global and hippocampal part of the cingulum, a region closely associated with the neurodegenerative processes of AD. In contrast, no significant associations were found between the RNFL and the DTI variables in the control region located outside the visual pathway and the regions involved in AD. No significant associations were found with global MRI variables.

Previous studies reported the associations between a thinner RNFL and lesions in the visual pathway in patients with multiple sclerosis,^6^ visual dysfunction,^28^ and glaucoma.^29,30,31^ Our results add complementary knowledge; we found an association between the RNFL thickness and the DTI variables in the posterior thalamic radiations, which include the visual pathway, not specifically in patients with diseases that directly influence the RNFL thickness but in a general elderly population. Therefore, even beyond specific pathologies inducing RNFL lesions, the RNFL may reflect the brain’s visual pathway. To our knowledge, only 2 previous studies^12,13^ have explored the association between the RNFL and brain MRI beyond the context of specific pathologies. The study by Ong et al^12^ included a population of 164 elderly participants and showed no association between the RNFL and the occipital lobe, which includes the visual cortex. However, only the occipital volume and not the DTI variables were studied. Moreover, that population differed from ours, with 75% of the participants being cognitively impaired; therefore, it was far from a general elderly population. The study by Casaletto et al,^13^ including 79 neurologically healthy older adults without global cognitive impairment (median Mini-Mental State Examination score, 29.5), focused on the mediotemporal lobe and did not analyze the association with the visual pathway.

In addition to the expected association with the visual pathway, our study showed that the RNFL thickness was associated with the HF and with the DTI variables in the global and hippocampal part of the cingulum, 2 brain structures that are involved in the neurodegenerative processes of AD. In contrast, no significant associations were found between the RNFL thickness and the DTI variables in the control region, which was located outside the visual pathway and the regions involved in AD. The hippocampus is well known to be involved early in the neurodegenerative processes, as shown in patients with MCI^32^ and even in cognitively intact elderly people before they developed AD.^29^ In previous results from the Three-City Study,^30^ it was found that people who later developed AD were initially seen with mediotemporal lesions up to 5 years before the diagnosis. The DTI variables in the WM structures have also been associated with current or future clinical neurodegenerative states. Compared with healthy controls, significant alterations in FA, RD, and MD have been shown in the parahippocampal cingulum of patients with AD and patients with MCI who convert to AD.^31^ Compared with stable healthy people, FA alterations were also detected in the parahippocampal cingulum and in the fornix in cognitively healthy people who developed amnestic MCI 2 years later.^33^ An association between glaucoma and AD has previously been found,^34,35,36^ although some work failed to confirm it.^37^ Our present results, which show that even in people without glaucoma, abnormal RNFL thinning is associated with brain abnormalities in regions involved in the neurodegenerative processes of AD, are in favor of common neurodegenerative processes occurring at the retinal and brain level, leading to both glaucoma and AD.

In our elderly population, which included people who were not specifically selected based on cognitive capacity, the associations identified between the RNFL and brain structures outside the visual pathway suggest a potential use for the RNFL to reflect brain alterations in structures that are particularly vulnerable in cognitive decline and AD. Concordant results were reported by Ong et al^12^ in their population with cognitive impairment and showed an association between the RNFL and the GM volume in the temporal lobe. In addition, Casaletto et al^13^ showed a specific association with the mediotemporal lobe.

We found no significant associations between the RNFL and global brain MRI variables. One explanation may be that our population was composed of generally well-functioning people; therefore, few participants had global alterations in the brain. In a previous MRI study^38^ of a small group of young people without multiple sclerosis (controls for patients with multiple sclerosis), the RNFL thickness was also not associated with BPF or GM and WM volumes. Moreover, Ong et al^12^ also found no association with the total brain or GM or WM volumes in their population with cognitive impairment; even in that population, an association was found only with the GM in the temporal lobe and not globally. However, before the moderate stage of dementia, global brain structures are preserved.

Therefore, associations with the RNFL seem to be specific to brain structures that are particularly vulnerable in the elderly population and are involved in the neurodegenerative processes of cognitive decline and AD. Regardless of the MRI variables, our results were consistent: a thicker RNFL was associated with both better DTI variables in the global and hippocampal part of the cingulum and a higher HF. A thicker RNFL was associated with lower RD and MD in the hippocampal part of the cingulum but was not associated with AxD. The RD may better reflect the dysmyelination of neurons, whereas AxD may better reflect axonal integrity.^39^ Previous studies^40,41^ have consistently found decreased microstructural integrity in WM tracts of patients with AD or at risk of AD, particularly in parahippocampal regions, with alterations in RD variables, suggesting that demyelination may be a contributing mechanism. Moreover, only the cingulum bundle and not the fornix was found to be associated with RNFL. Although the finding needs to be confirmed, this association only with the main afferent and not the efferent pathway of the hippocampus may correspond to an early stage of alteration. Therefore, our results may reflect an “early, silent” form of brain damage that may subsequently progress to a clinical stage. Casaletto et al^13^ previously found that the RNFL was associated with the mediotemporal lobe but not with memory performance and concluded that the RNFL may represent an early marker of AD processes. Within the Alienor Study, it was also previously found that a thinner RNFL was associated with lower delayed memory scores but not with other cognitive scores (including the Mini-Mental State Examination score), concordant with early stages of the neurodegenerative process.^42^

### Strengths and Limitations

This study has several strengths. It included a large population-based sample of elderly people who underwent exhaustive eye examinations, which allowed the exclusion of ocular pathologies that would likely have influenced the retinal measurements. The SD-OCT assessments were performed on the same SD-OCT machine by the same experienced technician according to a standardized protocol. Moreover, the spectral-domain technique provides the best quality and reproducibility currently available. The brain MRI measurements were also performed on the same machine and according to a standardized procedure. Two different types of MRI variables were analyzed (brain volumes and DTI variables) in different regions of the brain, allowing a better understanding of the global processes. Finally, our analyses accounted for numerous potential confounding factors.

Our study has some limitations. First, our population was composed of well-functioning people (who were well educated and had a conserved cognitive state) who accepted imaging and ophthalmological examinations; these participants differed from those without these examinations. These participants may have had fewer WM microstructural lesions, which may have prevented the detection of early MRI changes associated with the RNFL. However, to our knowledge, this is the first study to explore this association in a well-functioning population without dementia. Second, our cross-sectional design prevented the evaluation of any temporal association between retinal and brain degeneration; this would require a future longitudinal approach to determine if one of these degenerative processes precedes the other or if they are concurrent. Third, the analyses were explanatory and did not deal with multiple comparisons. Fourth, because our study was restricted to participants with both brain MRIs and ophthalmological examinations, we cannot exclude a possible lack of power.

## Conclusions

In elderly individuals without dementia, the RNFL thickness was associated with MRI variables in both regions that included the visual pathways and regions involved in AD-related neurodegeneration. Therefore, SD-OCT assessment of retinal axonal thickness may offer a way to explore brain MRI abnormalities in these regions. However, more studies are needed to demonstrate the potential utility of the RNFL thickness for detecting early brain neurodegeneration in presymptomatic elderly adults.

## Notes

Supplement. **eTable.** Comparison of the Characteristics According to the Inclusion in the Study Sample
