# Supplementary material for: Association of Retinal Nerve Fiber Layer Thickness With Brain Alterations in the Visual and Limbic Networks in Elderly Adults Without Dementia
Source: JAMA Netw Open. 2018 Nov 9;1(7):e184406. doi: 10.1001/jamanetworkopen.2018.4406 (PMC6324371; doi:10.1001/jamanetworkopen.2018.4406)
Supplement: Supplement. — eTable. Comparison of the Characteristics According to the Inclusion in the Study Sample [file jamanetwopen-1-e184406-s001.pdf]

Supplementary Online Content

Méndez-Gómez JL, Pelletier A, Rougier MB, et al. Association of retinal nerve fiber layer thickness with brain alterations in the visual and limbic networks in elderly adults without dementia. *JAMA Netw Open*. 2018;1(7):e184406. doi:10.1001/jamanetworkopen.2018.4406

eTable. Comparison of the Characteristics According to the Inclusion in the Study Sample

This supplementary material has been provided by the authors to give readers additional information about their work.

© 2018 Méndez-Gómez JL et al. *JAMA Network Open.*

eTable 1. Comparison of the characteristics according to the inclusion in the study sample

Socio-demographic, medical and brain imaging characteristics	"Volume" sample (n=104)*	3C population not included in the study sample (n=1110)**	*P* value[a]
Age (years), mean (SD)	80.8 (3.9)	83.4 (4.7)	<.0001
Female, n (%)	59 (56.7)	752 (67.0)	0.0225
Educational level, n (%)			
Elementary level	9 (8.7)	132 (11.9)	0.0163
Short secondary school	40 (38.5)	550 (49.6)	
Higher level	55 (52.9)	427 (38.5)	
Apolipoprotein E4, n (%)	16 (16.0)	182 (18.4)	0.5528
Diabetes, n (%)	9 (8.7)	148 (13.3)	0.1739
Hypertension, n (%)	76 (73.1)	935 (87.0)	0.0002
Smokers	36 (34.6)	370 (33.3)	0.7910
MMSE,[b] mean (SD)	27.7 (2.2)	26.3 (3.9)	<.0001

Abbreviations: MMSE, Mini Mental State Examination; SD, standard deviation.

*Missing data in volume sample: Apolipoprotein E4=4; MMSE= 1
**Missing data in 3C sample: Educational level= 1; Apolipoprotein E4=121; Hypertension= 35; MMSE= 52

[a]Chi2 test and Student ttest for quantitative and qualitative variables respectively
[b]The score ranges from 0 to 30, higher scores indicating better cognition

© 2018 Méndez-Gómez JL et al. *JAMA Network Open.*
